# Supplementary figures and images for: A Database of Lung Cancer-Related Genes for the Identification of Subtype-Specific Prognostic Biomarkers
Source: Biology (Basel). 2023 Feb 24;12(3):357. doi: 10.3390/biology12030357 (PMC10045015; doi:10.3390/biology12030357)

Logrank Test P-Value: 1.130e-4

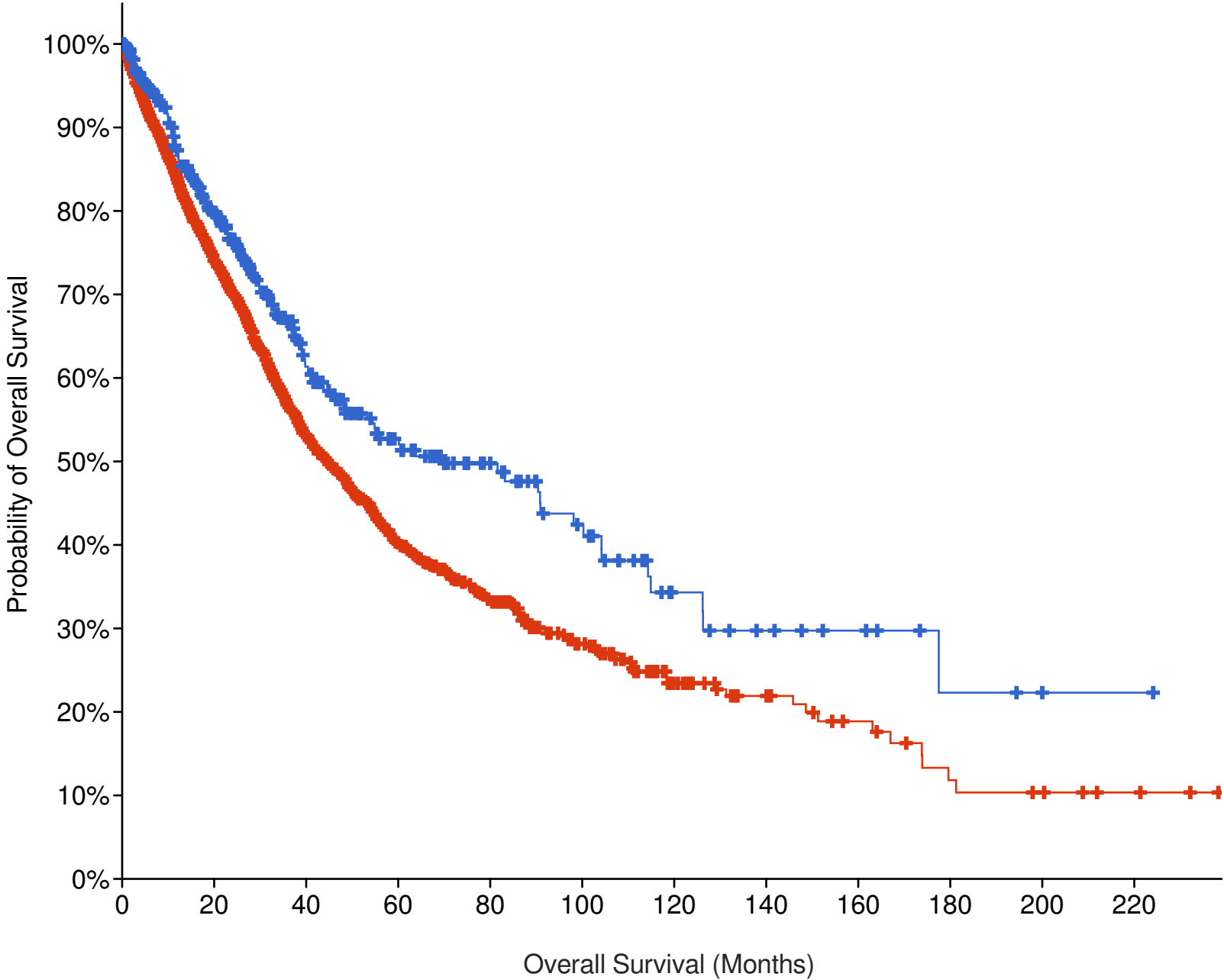

Overall

- Altered group
- Unaltered group

Supplement: Supplementary file 1 [file biology-12-00357-s001.zip › Figure S1. The overall survival analysis of 52 common genes shared in LUAD and LUSC.pdf]
